# Supplementary material for: The Use of Unique, Environmental Lactic Acid Bacteria Strains in the Traditional Production of Organic Cheeses from Unpasteurized Cow’s Milk
Source: Molecules. 2022 Feb 7;27(3):1097. doi: 10.3390/molecules27031097 (PMC8838525; doi:10.3390/molecules27031097)
Supplement: Supplementary file 1 [file molecules-27-01097-s001.zip › molecules-1552576-supplementary.pdf]

Supplementary Materials: The Use of Unique, Environmental Lactic Acid Bacteria Strains in the Traditional Production of Organic Cheeses from Unpasteurized Cow's Milk

Anna Łepecka [1,*], Anna Okoń [1], Piotr Szymański [1], Dorota Zielińska [2], Katarzyna Kajak-Siemaszko [2], Danuta Jaworska [2], Katarzyna Neffe-Skocińska [2], Barbara Sionek [2], Monika Trząskowska [2], Danuta Kołożyn-Krajewska [2] and Zbigniew J. Dolatowski [1]

[1] Department of Meat and Fat Technology, Prof. Waclaw Dabrowski Institute of Agriculture and Food, Biotechnology—State Research Institute,02-532 Warsaw, Poland; anna.okon@ibprs.pl (A.O.); piotr.szymanski@ibprs.pl (P.S.); zbigniew.dolatowski@ibprs.pl (Z.J.D.)

[2] Department of Food Gastronomy and Food Hygiene, Institute of Human Nutrition Sciences, University of Life Sciences-SGGW, 02-776 Warsaw, Poland; dorota_zielinska@sggw.edu.pl (D.Z.); katarzyna_kajak_siemaszko@sggw.edu.pl (K.K.-S.); danuta_jaworska@sggw.edu.pl (D.J.); katarzyna_neffe_skocinska@sggw.edu.pl (K.N.-S.); barbara_sionek@sggw.edu.pl (B.S.); monika_trzaskowska@sggw.edu.pl (M.T.); danuta_kolozyn_krajewska@sggw.edu.pl (D.K.-K.)

* Correspondence: anna.lepecka@ibprs.pl; Tel.: +48-225097025

Table S1. Fatty acid profiles in the tested cow cheeses after production and after 1 and 2 months of storage.

Fatty acid	Cheese Symbol								
	AW			B1			Os2		
4:0	2.33 ± 0.05 ^{bA}	2.58 ± 0.13 ^{aB}	2.38 ± 0.05 ^{aA}	2.45 ± 0.06 ^{cA}	2.63 ± 0.05 ^{aB}	2.35 ± 0.06 ^{aA}	2.28 ± 0.05 ^{aA}	2.60 ± 0.00 ^{aC}	2.40 ± 0.00 ^{aB}
6:0	1.50 ± 0.00 ^{bA}	1.58 ± 0.05 ^{aA}	1.58 ± 0.05 ^{bA}	1.53 ± 0.05 ^{bA}	1.63 ± 0.05 ^{aA}	1.58 ± 0.05 ^{bA}	1.45 ± 0.06 ^{aA}	1.60 ± 0.00 ^{aA}	1.50 ± 0.00 ^{aA}
8:0	0.90 ± 0.00 ^{aA}	0.90 ± 0.00 ^{aA}	1.00 ± 0.00 ^{aA}	0.90 ± 0.00 ^{aA}	0.90 ± 0.00 ^{aA}	1.00 ± 0.00 ^{aA}	0.85 ± 0.06 ^{aA}	0.90 ± 0.00 ^{aA}	0.90 ± 0.00 ^{aA}
10:0	2.00 ± 0.00 ^{aA}	2.10 ± 0.08 ^{aA}	2.28 ± 0.05 ^{aB}	2.00 ± 0.00 ^{aA}	2.10 ± 0.00 ^{aA}	2.20 ± 0.00 ^{aB}	1.95 ± 0.06 ^{aA}	2.15 ± 0.06 ^{aB}	2.20 ± 0.00 ^{aB}
10:1	0.20 ± 0.00 ^{aB}	0.13 ± 0.05 ^{aA}	0.20 ± 0.00 ^{aB}	0.20 ± 0.00 ^{aA}	0.10 ± 0.00 ^{aA}	0.20 ± 0.00 ^{aA}	0.20 ± 0.00 ^{aA}	0.20 ± 0.00 ^{bA}	0.20 ± 0.00 ^{aA}
12:0	2.45 ± 0.06 ^{bA}	2.58 ± 0.10 ^{aB}	2.68 ± 0.05 ^{aB}	2.43 ± 0.05 ^{bA}	2.55 ± 0.06 ^{aA}	2.70 ± 0.00 ^{aB}	2.35 ± 0.06 ^{aA}	2.55 ± 0.06 ^{aB}	2.60 ± 0.00 ^{aB}
12:1	0.10 ± 0.00 ^{aA}	0.10 ± 0.00 ^{aA}	0.10 ± 0.00 ^{aA}	0.10 ± 0.00 ^{aA}	0.10 ± 0.00 ^{aA}	0.10 ± 0.00 ^{aA}	0.10 ± 0.00 ^{aA}	0.10 ± 0.00 ^{aA}	0.10 ± 0.00 ^{aA}
13:0	0.10 ± 0.00 ^{aA}	0.10 ± 0.00 ^{aA}	0.10 ± 0.00 ^{aA}	0.10 ± 0.00 ^{aA}	0.10 ± 0.00 ^{aA}	0.10 ± 0.00 ^{aA}	0.10 ± 0.00 ^{aA}	0.10 ± 0.00 ^{aA}	0.10 ± 0.00 ^{aA}
14:0	9.75 ± 0.06 ^{aA}	9.80 ± 0.08 ^{aA}	9.83 ± 0.05 ^{aA}	9.90 ± 0.00 ^{bA}	9.98 ± 0.05 ^{bA}	10.05 ± 0.06 ^{bA}	9.78 ± 0.05 ^{aA}	9.78 ± 0.05 ^{aA}	9.88 ± 0.05 ^{aA}
14:1	1.03 ± 0.05 ^{aA}	1.10 ± 0.00 ^{aA}	1.10 ± 0.00 ^{aA}	1.10 ± 0.00 ^{bA}	1.10 ± 0.00 ^{aA}	1.20 ± 0.00 ^{aA}	1.08 ± 0.05 ^{bA}	1.10 ± 0.00 ^{aA}	1.10 ± 0.00 ^{aA}
15:0 br	0.70 ± 0.00 ^{aA}	0.78 ± 0.05 ^{aA}	0.80 ± 0.00 ^{aA}	0.70 ± 0.00 ^{aA}	0.75 ± 0.06 ^{aA}	0.80 ± 0.00 ^{aA}	0.70 ± 0.00 ^{aA}	0.78 ± 0.05 ^{aA}	0.80 ± 0.00 ^{aA}
15:0	1.20 ± 0.00 ^{aA}	1.23 ± 0.05 ^{aA}	1.30 ± 0.00 ^{aA}	1.20 ± 0.00 ^{aA}	1.30 ± 0.00 ^{bA}	1.35 ± 0.06 ^{aA}	1.20 ± 0.00 ^{aA}	1.30 ± 0.00 ^{bA}	1.35 ± 0.06 ^{aA}
15:1	0.30 ± 0.00 ^{aA}	0.30 ± 0.00 ^{aA}	0.30 ± 0.00 ^{aA}	0.30 ± 0.00 ^{aA}	0.30 ± 0.00 ^{aA}	0.30 ± 0.00 ^{aA}	0.30 ± 0.00 ^{aA}	0.30 ± 0.00 ^{aA}	0.30 ± 0.00 ^{aA}
16:0	24.55 ± 0.06 ^{aB}	24.40 ± 0.12 ^{aA}	24.28 ± 0.05 ^{aA}	25.13 ± 0.05 ^{bA}	25.15 ± 0.06 ^{bA}	25.00 ± 0.00 ^{cA}	25.03 ± 0.15 ^{bB}	24.70 ± 0.14 ^{aA}	24.78 ± 0.10 ^{bA}
16:1	1.53 ± 0.05 ^{aA}	1.60 ± 0.00 ^{aA}	1.70 ± 0.00 ^{aB}	1.55 ± 0.06 ^{aA}	1.70 ± 0.00 ^{bB}	1.70 ± 0.00 ^{aB}	1.60 ± 0.00 ^{bA}	1.75 ± 0.06 ^{bB}	1.78 ± 0.05 ^{aB}
17:0 br	0.90 ± 0.00 ^{aA}	0.90 ± 0.00 ^{aA}	1.00 ± 0.00 ^{aA}	0.90 ± 0.00 ^{aA}	0.90 ± 0.00 ^{aA}	1.00 ± 0.00 ^{aA}	0.90 ± 0.00 ^{aA}	0.90 ± 0.00 ^{aA}	1.00 ± 0.00 ^{aA}
17:0	0.60 ± 0.00 ^{aA}	0.60 ± 0.00 ^{aA}	0.70 ± 0.00 ^{aA}	0.60 ± 0.00 ^{aA}	0.60 ± 0.00 ^{aA}	0.70 ± 0.00 ^{aA}	0.60 ± 0.00 ^{aA}	0.60 ± 0.00 ^{aA}	0.70 ± 0.00 ^{aA}
17:1	0.20 ± 0.00 ^{aA}	0.20 ± 0.00aA	0.20 ± 0.00 ^{aA}	0.20 ± 0.00 ^{aA}	0.20 ± 0.00 ^{aA}	0.20 ± 0.00 ^{aA}	0.20 ± 0.00 ^{aA}	0.20 ± 0.00 ^{aA}	0.20 ± 0.00 ^{aA}
18:0	11.45 ± 0.06 ^{bB}	11.33 ± 0.05 ^{bA}	11.35 ± 0.06 ^{bA}	11.0 ± 0.00 ^{aA}	10.83 ± 0.05 ^{aA}	10.93 ± 0.05 ^{aA}	11.03 ± 0.05 ^{aB}	10.68 ± 0.10 ^{aA}	10.90 ± 0.00 ^{aB}

18:1 trans	7.08 ± 0.05 ^{cA}	7.03 ± 0.05 ^{cA}	7.10 ± 0.00 ^{bA}	6.73 ± 0.05 ^{aA}	6.73 ± 0.05 ^{aA}	6.80 ± 0.08 ^{aA}	6.95 ± 0.06 ^{bA}	6.90 ± 0.00 ^{bA}	7.08 ± 0.05 ^{bB}
18:1 cis9	20.83 ± 0.13 ^{aB}	20.80 ± 0.08 ^{aB}	20.38 ± 0.15 ^{bA}	20.83 ± 0.05 ^{aB}	20.70 ± 0.14 ^{aB}	20.23 ± 0.17 ^{aA}	21.08 ± 0.15 ^{bB}	20.85 ± 0.06 ^{aB}	20.35 ± 0.06 ^{bA}
18:1 cis11	0.88 ± 0.05 ^{bA}	0.80 ± 0.00 ^{aA}	0.80 ± 0.00 ^{aA}	0.90 ± 0.00 ^{bA}	0.80 ± 0.00 ^{aA}	0.80 ± 0.00 ^{aA}	0.80 ± 0.00 ^{aA}	0.80 ± 0.00 ^{aA}	0.80 ± 0.00 ^{aA}
18:1	1.70 ± 0.00 ^{aA}	1.65 ± 0.06 ^{aA}	1.70 ± 0.00 ^{aA}	1.75 ± 0.06 ^{aA}	1.68 ± 0.05 ^{aA}	1.68 ± 0.05 ^{aA}	1.65 ± 0.06 ^{aA}	1.70 ± 0.00 ^{aA}	1.70 ± 0.00 ^{aA}
18:2	2.55 ± 0.10 ^{aB}	2.08 ± 0.10 ^{bA}	1.98 ± 0.05 ^{aA}	2.50 ± 0.08 ^{aB}	1.88 ± 0.05 ^{aA}	1.90 ± 0.00 ^{aA}	2.58 ± 0.10 ^{aB}	1.95 ± 0.10 ^{aA}	1.90 ± 0.00 ^{aA}
18:3 n3	1.40 ± 0.00 ^{aA}	1.50 ± 0.00 ^{aA}	1.40 ± 0.00 ^{aA}	1.40 ± 0.00 ^{aA}	1.40 ± 0.00 ^{aA}	1.40 ± 0.00 ^{aA}	1.40 ± 0.00 ^{aA}	1.50 ± 0.00 ^{aA}	1.40 ± 0.00 ^{aA}
18:2 c9t11	2.63 ± 0.05 ^{aA}	2.78 ± 0.05 ^{aB}	2.60 ± 0.00 ^{aA}	2.63 ± 0.05 ^{aA}	2.75 ± 0.06 ^{aA}	2.65 ± 0.06 ^{aA}	2.70 ± 0.00 ^{bA}	2.85 ± 0.06 ^{bA}	2.80 ± 0.00 ^{bA}
20:0	0.10 ± 0.00 ^{aA}	0.10 ± 0.00 ^{aA}	0.10 ± 0.00 ^{aA}	0.10 ± 0.00 ^{aA}	0.10 ± 0.00 ^{aA}	0.10 ± 0.00 ^{aA}	0.10 ± 0.00 ^{aA}	0.10 ± 0.00 ^{aA}	0.10 ± 0.00 ^{aA}
20:1	0.30 ± 0.00 ^{bA}	0.30 ± 0.00 ^{aA}	0.30 ± 0.00 ^{aA}	0.25 ± 0.06 ^{aA}	0.30 ± 0.00 ^{aA}	0.30 ± 0.00 ^{aA}	0.30 ± 0.00 ^{bA}	0.30 ± 0.00 ^{aA}	0.30 ± 0.00 ^{aA}
20:2	0.10 ± 0.00 ^{aA}	0.10 ± 0.00 ^{aA}	0.10 ± 0.00 ^{aA}	0.10 ± 0.00 ^{aA}	0.10 ± 0.00 ^{aA}	0.10 ± 0.00 ^{aA}	0.10 ± 0.00 ^{aA}	0.10 ± 0.00 ^{aA}	0.10 ± 0.00 ^{aA}
20:3 n6	0.10 ± 0.00 ^{aA}	0.10 ± 0.00 ^{aA}	0.10 ± 0.00 ^{aA}	0.10 ± 0.00 ^{aA}	0.10 ± 0.00 ^{aA}	0.10 ± 0.00 ^{aA}	0.10 ± 0.00 ^{aA}	0.10 ± 0.00 ^{aA}	0.10 ± 0.00 ^{aA}
20:4 n6	0.10 ± 0.00 ^{aA}	0.10 ± 0.00 ^{aA}	0.10 ± 0.00 ^{aA}	0.10 ± 0.00 ^{aA}	0.10 ± 0.00 ^{aA}	0.10 ± 0.00 ^{aA}	0.10 ± 0.00 ^{aA}	0.10 ± 0.00 ^{aA}	0.10 ± 0.00 ^{aA}
22:0	0.10 ± 0.00 ^{aA}	0.10 ± 0.00 ^{aA}	0.10 ± 0.00 ^{aA}	0.10 ± 0.00 ^{aA}	0.10 ± 0.00 ^{aA}	0.10 ± 0.00 ^{aA}	0.10 ± 0.00 ^{aA}	0.10 ± 0.00 ^{aA}	0.10 ± 0.00 ^{aA}
22:4 n6	0.10 ± 0.00 ^{aA}	0.10 ± 0.00 ^{aA}	0.10 ± 0.00 ^{aA}	0.10 ± 0.00 ^{aA}	0.10 ± 0.00 ^{aA}	0.10 ± 0.00 ^{aA}	0.10 ± 0.00 ^{aA}	0.10 ± 0.00 ^{aA}	0.10 ± 0.00 ^{aA}
22:5 n3	0.10 ± 0.00 ^{aA}	0.10 ± 0.00 ^{aA}	0.10 ± 0.00 ^{aA}	0.10 ± 0.00 ^{aA}	0.18 ± 0.05 ^{bA}	0.10 ± 0.00 ^{aA}	0.10 ± 0.00 ^{aA}	0.18 ± 0.05 ^{bB}	0.10 ± 0.00 ^{aA}
	0	1	2	0	1	2	0	1	2
	Time (month)								

AW, cheese with acid whey; B1, cheese with *Levilactobacillus brevis* B1; Os2, cheese with *Lactiplantibacillus plantarum* Os2. The values are expressed as means ±SD, means in the same row followed by different uppercase letters (^{A}–^{C}) within the same sample in different times are significantly different ($p < 0.05$), means in the same column followed by different lowercase letters (^{a}–^{c}) within samples in the same time are significantly different ($p < 0.05$), $n = 4$.
